# The Discriminant Use of Intrauterine Balloon Tamponade and Compression Sutures for Management of Major Postpartum Hemorrhage: Comparison of Patient Characteristics and Clinical Outcome

**DOI:** 10.1155/2021/6648829

**Published:** 2021-01-02

**Authors:** Choi Wah Kong, William Wing Kee To

**Affiliations:** Department of Obstetrics and Gynaecology, United Christian Hospital, Hong Kong

## Abstract

**Background:**

Intrauterine balloon tamponade (IUBT) and compression sutures have been widely used in recent years in the management of postpartum hemorrhage (PPH). However, there is scant literature directly comparing the clinical scenarios that led to the discriminant selection of these management modalities and the direct clinical outcomes. The purpose of this study is to compare the patient characteristics and clinical risk factors that led to the use of IUBT and compression sutures in the management of major PPH as well as the immediate outcome in a retrospective cohort.

**Methods:**

Patients who had IUBT or compression sutures applied due to major PPH (>1000 ml) from 2014 to 2018 in a single obstetric unit were recruited. The patient characteristics and clinical outcome of the two groups were compared.

**Results:**

A total of 67 patients had IUBT and 29 patients had compression sutures applied as the first uterine sparing technique. Apart from more vaginal deliveries (25.4% vs. 3.5%) in the IUBT group compared to compression sutures, there were no significant differences between the two groups in terms of patient characteristics. The IUBT group had a slightly higher blood loss at the start of the uterine sparing procedure (239 ml, *p* = 0.049) and received more transfusions, despite no differences in the total blood loss, hemogloblin level, incidence of coagulopathy, and intensive care unit admission between the two groups. There was no significant difference in the overall success rate between IUBT and compression sutures to control PPH without additional surgical intervention or hysterectomy (73.1% vs. 55.1%, *p* = 0.15) or the success rate for PPH due to uterine atony (32.8% vs. 20.7%), though IUBT apparently performed better than compression sutures in cases of placenta praevia (77.3% vs. 16.7%, *p* = 0.01). Blood loss > 1.5 l at the start of the procedure, presence of placenta accreta, and presence of coagulopathy were found to be significant poor prognostic factors for both procedures to control PPH.

**Conclusions:**

There were no dominating patient characteristics that favoured the selection of either IUBT or compression sutures in the management of severe PPH except for the mode of delivery. Both procedures had equally high overall success rates to control PPH, but IUBT performed better in placenta praevia cases as compared to compression sutures.

## 1. Introduction

Postpartum hemorrhage (PPH) is one of the leading causes of maternal mortality. The basic treatment of PPH consists of medical management by uterotonic drugs such as oxytocin, and prostaglandin or their analogues. Traditionally, peripartum hysterectomy would be performed in patients with massive PPH who failed medical treatment. Various uterine sparing procedures have been developed in recent years to reduce the need for hysterectomy, including intrauterine balloon tamponade, uterine compression sutures, selective devascularization by surgical ligation, or radiological embolization of the uterine and pelvic arteries [1–3]. In recent decades, these second-line conservative surgical procedures have been gradually incorporated into protocols for severe PPH management [4].

The first case report of uterine compression sutures was published in 1996 with a single patient from Zurich [5]. B-Lynch et al. published a case report of five consecutive cases utilizing the B-Lynch suture in 1997 [6]. Various modifications of the B-Lynch suture and various other compression suture techniques have been reported since then. However, the B-Lynch suture remained the most widely performed suture among all the compression sutures [7]. On the other hand, the intrauterine balloon tamponade had been attempted for years using Foley catheters, Sengstaken-Blakemore balloons, or other adaptations. Bakri advocated the use of a uterine-specific silicone Bakri balloon since 2001. From then on, the Bakri balloon and other uterine-specific balloon tamponade systems had been widely used as second-line management for PPH.

So far, there were no randomized control trials to directly compare the efficacy of the two treatment modalities in the literature. Understandably, such trials were considered impossible or impractical under the dire circumstances of severe postpartum hemorrhage, and the discriminant use of either modality of management is usually dependent on the preference and experience of the attending obstetrician. This study is aimed at comparing the clinical characteristics and risk factors of patients with major PPH undergoing these two widely performed second-line procedures to delineate any significant differences in patient selection, as well as their immediate clinical outcome.

## 2. Materials and Methods

This study consisted of a retrospective cohort of all patients with major PPH > 1000 ml from 2014 to 2018 (5-year period) in a single obstetric-training unit. PPH was managed in accordance with a standard protocol, starting with various oxytoxic agents including syntometrine, syntocinon bolus and infusion, and carboprost injections. When medical treatment failed to control hemorrhage, second-line conservative procedures by either IUBT or compression sutures would be used depending on the clinical situation and attending obstetrician's preference and experience. The amount of blood loss was quantified by measuring the blood loss in the suction bottle and weighing the abdominal pads and gauzes intraoperatively. The Bakri balloon was the only balloon tamponade system used in our unit during the study period. The procedure for application of the Bakri balloon was in accordance with that generally described in the literature [3, 8]. The Bakri balloon can be placed transvaginally through the cervix or transabdominally through the caesarean uterine wound depending on the route of delivery and as decided by the attending obstetrician. The B-Lynch suture was the only compression suture technique adopted in our unit during the study period and was performed largely as originally described by B-Lynch in 1997 [6]. Patients who failed medical treatment were categorized according to the first attempted uterine sparing technique (intention to treat analysis). Patients' clinical risk factors, aetiology of PPH, and the amount of blood loss at the time of utilization of these treatment modalities were compared. Outcomes were then categorized as successful (when no additional interventions were required), as additional procedures (when additional uterine sparing procedures including intrauterine balloon tamponade, compression sutures, radiological uterine arterial embolization, or surgical pelvic devascularization were required in addition to the primary procedure employed), and as hysterectomy (when hysterectomy was required). Secondary outcome parameters include total blood loss, coagulopathy, need for blood product transfusion, intensive care unit admission, need for relaparotomy, or other serious maternal complications or death. Secondary analysis was performed to evaluate the efficacy of IUBT and compression sutures performed in accordance with the major indications of PPH, namely, uterine atony or placenta praevia/accreta. Those with direct hysterectomy without attempting uterine sparing techniques were excluded from analysis. Those in which the uterine sparing procedures were applied in a prophylactic manner with total blood loss < 1000 ml were excluded from analysis.

Based on our previous audit data, around 75% of all patients with severe PPH were delivered by CS with 25% by vaginal delivery, and the aetiology of PPH was uterine atony in around 66.7% and nonatonic causes in the rest. Using the mode of delivery and aetiology of PPH as the key discriminating factors and assuming the correlation between the choice of procedure and either of these factors to be 40%, a sample size of around 69 and 49, respectively, would be required using a two-sided test at 5% significance level test (*α* = 0.05) with power 80% (*β* = 0.2). Similarly, assuming around 70% of patients would undergo IUBT and 30% would have compression sutures, a sample size of around 90 would be required to show a difference in success rate of around 30% between the two procedures using a two-sided test at similar significance level and power. With an average severe PPH rate estimated at around 1.5%, it was therefore decided that an analysis of all patients with severe PPH over a five-year period should provide a sufficient sample.

The obstetric data of all the above patients with PPH were identified from a comprehensive obstetric database. The electronic and paper records of these identified cases were then studied in detail. The SPSS for Windows package was used for data entry and analysis. Continuous variables were analyzed by *t*-test and discrete variables by chi-square test or Fisher's exact test when appropriate. A binary logistic regression model using the enter technique was constructed to evaluate the prognostic factors for success by including parameters found to be significant on univariate analysis. A *p* value of less than 0.05 (*p* < 0.05) was considered statistically significant. Ethical approval for this study was granted by the Kowloon Central/Kowloon East Ethics Committee Board of the Hospital Authority, Hong Kong. As this study was a retrospective review of patient outcome, patient consent was waived by the Ethics Committee Board.

## 3. Results

**Figure 1:**
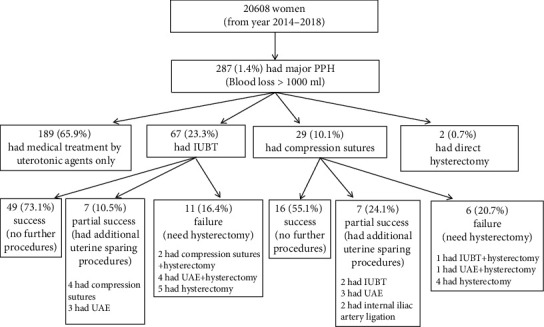
The treatment modalities that were adopted for the women with major postpartum hemorrhage in this cohort. PPH: postpartum hemorrhage; IUBT: intrauterine balloon tamponade; UAE: uterine artery embolization.

There were a total of 20608 deliveries in the study period. The incidence of PPH (>500 ml) was 1529/20608 (7.4%) while the incidence of major PPH (>1000 ml) was 287/20608 (1.4%). Among those with major PPH, 189 (65.9%) were successfully managed by medical treatment alone, 67 (23.3%) had IUBT as the first uterine sparing technique after failed medical treatment, and 29 (10.1%) had compression sutures as the first uterine sparing technique while 2 (0.7%) had direct hysterectomy performed without attempting any uterine sparing procedures. The results are shown in Figure 1.

Comparing patient characteristics, there were no differences in the maternal demographic data such as maternal age, parity, and gestation at delivery between the IUBT group and the compression suture group. There was also no difference in the aetiology of PPH in the start of the uterine sparing procedure between the two groups. There were more vaginal deliveries in the IUBT group while there were more CS deliveries in the compression suture group. As the use of compression sutures would require additional laparotomy in these patients after vaginal deliveries, IUBT would logically be preferred as the first uterine sparing technique instead of laparotomy. There were more twin pregnancies in the IUBT group, despite the fact that all were delivered by CS, but the difference was not statistically significant. The blood loss at the start of the uterine sparing procedure was higher in the IUBT group compared with the compression suture group (1305 vs. 1117 ml, mean difference 239 ml, *p* = 0.049), while the rate of transfusion of blood products in the IUBT group was also higher than that in the compression suture group, despite there being no differences in the total blood loss, lowest hemogloblin level, incidence of coagulopathy, and intensive care unit admission between the two groups (Table 1).

For the group having IUBT as the first uterine sparing technique, the success rate was 73.1% (49/67) with the bleeding controlled without further surgical intervention, while 10.5% (7/67) required additional uterine sparing procedures, and 16.4% (11/67) required hysterectomy. For the group having compression sutures as the first uterine sparing technique, the success rate was 55.1% (16/29), additional procedures were required in 24.1% (7/29), and hysterectomy was required in 20.7% (6/29). Specifically, in the IUBT group, concurrent compression sutures were applied in 3 cases, and similarly, in the compression suture group, IUBT was also applied simultaneously in 3 cases, so that in total, 6 cases underwent the “sandwich” procedure. There were no statistically significant differences between the success rate, need for additional procedures, and hysterectomy between the two groups (*p* = 0.15) for all severe PPH cases, as well as for PPH due to uterine atony (*p* = 0.09). However, IUBT apparently performed better than compression sutures for placenta praevia/accreta cases when analyzing the overall success rate (17/22, 77.3% vs. 1/6, 16.7%, *p* = 0.01) (Table 2).

Within the cohort, five women were diagnosed with placenta accreta prenatally while one had placenta accreta diagnosed intraoperatively during CS. All of them had a balloon tamponade inserted, but only one of them was successfully managed with the balloon tamponade; the other five women finally required hysterectomy (including the woman having placenta accreta diagnosed intraoperatively). Placenta accreta was confirmed in all five women who underwent hysterectomy on subsequent histopathological examination of the uterus.

Evaluating the risk factors for need for additional procedures and hysterectomy after use of either IUBT or compression sutures, blood loss > 1.5 l at the start of the procedure, presence of placenta accreta, and presence of coagulopathy were found to be significant in univariate analysis as well as after logistic regression analysis (Tables 3 and 4).

## 4. Discussion

While we were able to demonstrate an equal and high overall success rate for both IUBT and compression sutures in the management of severe PPH, the main limitation of this study is that the selection of which uterine sparing procedure to apply would depend on the patient's clinical condition, obstetrician's preference and experience, or whether a laparotomy was already performed for CS. Randomized controlled trials appear to be impractical to be conducted in such dire obstetric emergencies, and standardization of the selection criteria for which procedure to use would also be extremely difficult. However, despite the possible biases in the choice of uterine sparing procedure, our data showed no evidence of any compelling patient characteristics or clinical parameter that would preclude or greatly favour the use of one procedure over the other apart from the mode of delivery. IUBT was preferred for severe PPH after vaginal delivery as compared to compression sutures, obviously because of the intention to avoid a laparotomy. While the blood loss at the start of the uterine sparing procedure was higher for the IUBT group by a mean value of 239 ml, the difference was likely to be coincidental and might not have any direct impact on the clinical outcome. We believe that our data would therefore demonstrate the general experience of the use of these techniques in real-life situations in the labour ward, notwithstanding the limitations posed by a retrospective cohort where the choice of procedure was entirely the decision of the attending obstetrician.

In a systematic review of 46 studies [2], the success rate of the balloon tamponade was 84% and for uterine compression sutures was 91.7%. However, this review consists of studies which investigated the use of IUBT and compression sutures separately in different centers. Currently, there is very scarce data showing head-on comparison for these two modalities in the same centers. A retrospective cohort published in 2016 investigated 45 women having uterine atony during CS. The success rate of IUBT to stop bleeding alone without additional procedures did not differ from compression sutures (80.0% versus 79.1%, *p* = 0.76) [9]. Another retrospective cohort published in 2018 investigated 82 patients having uterine atony during CS. The success rate of IUBT to stop bleeding alone without additional procedures again did not differ from Hayman sutures (74.4% versus 76.7%, *p* = 0.80) [10]. However, as with our current cohort, the decision for either procedure in these two studies also rested entirely on the attending obstetrician. We have attempted to compare directly the clinical outcome of IUBT and compression sutures in severe PPH in both vaginal deliveries as well as in CS and included all major causes of PPH apart from uterine atony. We believe that such an evaluation should be valid and practical, simulating practical labour ward situations. While the overall success rate of IUBT to control major PPH without the need for additional procedures or peripartum hysterectomy in this cohort was apparently higher than that for compression sutures (73.1% vs. 55.1%), the difference did not reach statistical significance. In addition, the success rate of IUBT and compression sutures did not differ in PPH caused by uterine atony (*p* = 0.48) which was similar to the findings reported by the two studies quoted above.

Our data showed that in PPH caused by placenta praevia/accreta, the success rate of IUBT was much higher than that of compression sutures (*p* = 0.01). The cause for PPH in placenta praevia/accreta is often due to bleeding from the placental bed after delivery of the placenta. Apart from compressing the uterine body in uterine atony, the B-Lynch compression suture was also advocated to use for placenta praevia in its original description [6]. It was proposed that the sutures would exert longitudinal compression and achieve evenly distributed tension over the uterus including the lower segment, or additional independent figure-of-eight sutures could be placed on the lower segment [6]. Previous reviews on the general success rate for compression sutures have also reported a high rate even for placenta praevia cases [11, 12]. Hayman et al. had described placing two transverse sutures in the lower segment in addition to the Hayman compression sutures in order to control bleeding from placenta praevia [1]. Other reports have advocated various techniques for applying separate parallel vertical sutures over the lower segment without brace sutures to effectively compress the placental bed directly to stop bleeding from placenta praevia [13–15]. The application of these lower segment sutures would require additional surgical skills and might not be readily mastered by more junior obstetricians. In our cohort, traditional B-lynch sutures were applied with additional hemostatic stitches over the lower segment in only 2 of the 6 praevia cases, and this could contribute to the lower success rate for compression sutures compared with IUBT in managing this group of patients.

On the other hand, we would postulate that the use of the Bakri balloon could cause a direct pressure effect on the placental bed to promote hemostasis. Indeed, Bakri et al.'s original publication in 2001 described the use of the Bakri balloon in a case series of 5 patients with placenta praevia [3]. Subsequent studies found that the Bakri balloon was apparently effective in arresting bleeding caused by uterine atony despite the arguments against the “paradoxical” use of a balloon tamponade to expand rather than to contract the uterus in the presence of uterine atony [16, 17]. The use of IUBT was then gradually extended to all causes of PPH. As the application of IUBT should be technically much less demanding than compression sutures, it would logically be the preferred first choice surgical procedure in praevia/accreta cases.

The insertion of the Bakri balloon subsequent to the application of compression sutures was described as “uterine sandwich technique.” Nelson and O'Brien had reported using this technique in 5 women, and it was apparently effective in all of them [18]. Application of compression sutures when the Bakri balloon was already inserted in utero was called as the reverse sandwich technique. Diemert et al. had reported this technique on seven women and bleeding was controlled in 85.0% of them [19]. It remains controversial in the literature that when the sandwich techniques are indicated, whether compression sutures or IUBT should be performed first [9]. In our cohort, the success rates of the uterine sandwich technique and reverse sandwich technique were both 66.7% (uterine sandwich technique 2/3; reverse sandwich technique 2/3), with no differences between the sequence of procedure and the final success rate of arresting bleeding. In our experience, to apply compression sutures over the uterus with a fully inflated balloon inside the uterine cavity would be difficult, and the balloon could easily be punctured by the suture needle. We would therefore deflate the balloon to only 100 ml for application of the compression suture and reinflate the balloon afterwards. However, such deflation and reinflation of the Bakri balloon could take up considerable precious time, and one needs to be extremely vigilant on the amount of ongoing bleeding during the maneuver. On the other hand, inserting the Bakri balloon into an already compressed uterine cavity after completion of compression sutures could also be technically difficult, as the balloon might sometimes fail to be inserted or otherwise could easily slip out of the cervix afterwards.

While major complications had been reported after application of compression sutures including uterine necrosis, pyometra, and endometritis [20–22], there were no complications recorded in our cohort. Evidence for whether compression sutures could affect subsequent fertility remains conflicting. Some studies found no adverse effects [23, 24] while some found that fertility could be affected by the intrauterine synechiae and deformation in the uterine fundus caused by the sutures [22, 25]. Equally, there were no complications after insertion of the Bakri balloon in our cohort, though major morbidity resulting from uterine rupture due to migration of the balloon has been reported [26]. There appears to be an advantage for the use of the Bakri balloon concerning subsequent fertility, as we had previously reported that the Bakri balloon poses little adverse effects on subsequent reproductive function in a follow-up cohort after treatment with the balloon tamponade [27]. Uterine sandwich techniques have also been associated with major complications, such as delayed uterine necrosis that finally required hysterectomy [28]. It was therefore suggested that uterine blanching should be looked for when applying sandwich techniques which could signify inadequate blood flow to the uterus. We should be well aware of such rare complications when sandwich techniques are employed.

Our data demonstrated that blood loss > 1.5 l at the start of application of IUBT or compression sutures, placenta accreta, and presence of coagulopathy were significant poor prognostic factors for these uterine sparing procedures to control PPH. Previous studies had found that the earlier insertion of the balloon tamponade when the blood loss was less would lead to a higher success rate to control PPH without hysterectomy [29–31]. In addition, presence of coagulopathy was identified as an independent factor associated with failure in other studies [31, 32]. Although disseminated intravascular coagulopathy has been identified as a prognostic factor for the success of the balloon tamponade, the presence of coagulopathy did not imply that all women with coagulopathy would fail, as it had been demonstrated that over one-third of such cases would still be successful [31]. The presence of these prognostic factors could prompt the obstetricians to proceed to hysterectomy early in order to reduce total blood loss and the need for massive transfusion, as well as to ameliorate maternal morbidity and mortality.

## 5. Conclusion

Our data showed no discriminating patient or clinical parameter that would favour the use of IUBT or compression sutures in the management of severe PPH apart from the mode of delivery. IUBT and compression sutures had similar overall success rates to control severe PPH or, specifically, for cases caused by uterine atony, but the former performed better in placenta praevia cases. Blood loss > 1.5 l at the start of the procedure, presence of placenta accreta, and presence of coagulopathy were found to be significant poor prognostic factors for both procedures to control PPH.

## Figures and Tables

**Table 1:** Comparison of the characteristics between women treated with IUBT and those treated with compression sutures.

	IUBT (*n* = 67)	Compression sutures (*n* = 29)	*p* value
Age	33.3 (SD 4.3)	33.2 (SD 4.7)	0.92; MD 0.09 (CI -1.87 to 2.05)
Advanced maternal age (*age* ≥ 35)	29 (43.2%)	11 (37.9%)	0.39
Parity			
Nulliparous	33 (49.2%)	13 (44.8%)	0.43
Multiparous	34 (50.8%)	16 (55.2%)	
Gestation at delivery	38.2 (SD 1.7)	38.07 (SD 1.7)	0.74; MD 0.12 (CI -0.64 to 0.88)
*Preterm* *delivery* < 37 *weeks*	3 (4.5%)	3 (10.3%)	0.25
Multiple pregnancy	9 (13.4%)	2 (6.9%)	0.59
Previous CS			
One previous CS	7 (10.4%)	5 (17.2%)	0.64
Two previous CS	3 (4.5%)	1 (3.5%)	
Mode of delivery			
Normal vaginal	17 (25.4%)	1 (3.5%)	<0.05^∗^
Instrumental	2 (3.0%)	1 (3.5%)	
Caesarean section	48 (71.6%)	27 (93.1%)	
Principle indication for CS	*n* = 48	*n* = 27	
Placenta praevia	22 (45.8%)	6 (22.2%)	0.55
Abruptio placenta	2 (4.2%)	1 (3.7%)	
Previous CS	1 (2.0%)	1 (3.7%)	
Breech	4 (8.3%)	2 (7.4%)	
No progress of labour	8 (16.8%)	7 (26.0%)	
Multiple pregnancy	6 (12.5%)	1 (3.7%)	
Fetal distress	3 (6.2%)	5 (18.5%)	
Failed induction	0 (0.0%)	1 (3.7%)	
Maternal disease	0 (0.0%)	1 (3.7%)	
Prolonged second stage/failed instrumental delivery	2 (4.2%)	2 (7.4%)	
Aetiology of PPH			
Uterine atony	42 (62.7%)	23 (79.3%)	0.21
Placenta praevia/accreta	22 (32.8%)	6 (20.7%)	
Genital tract trauma	3 (4.5%)	0 (0.0%)	
Blood loss at start of uterine sparing procedure (ml)	1305 (SD 408) (range 800-2500)	1117 (SD 316) (range 800-2000)	0.049^∗^; MD 239 (CI 1.33 to 478)
Additional procedures			
IUBT	—	3 (10.3%)	
Compression sutures	6 (8.9%)	—	
UAE	7 (10.4%)	4 (13.8%)	
Pelvic devascularization	0 (0.0%)	2 (6.9%)	
Hysterectomy	11 (16.4%)	6 (20.7%)	
Total additional procedures	24 (35.8%)	15 (51.7%)	0.14
Coagulopathy	24 (35.8%)	8 (27.6%)	0.29
Total blood loss (ml)	2356 (SD 1550) (range 1100-8000)	2744 (SD 1980) (range 1150-9300)	0.30; MD -388 (CI -1134 to 357)
Lowest hemoglobin level (g/dl)	8.3 (SD 1.7) (3.6-12.9)	8.4 (SD 1.2) (5.6-10.1)	0.76; MD -0.11 (CI -0.82 to 0.60)
Transfusion of blood products	58 (86.5%)	19 (65.5%)	0.02^∗^
ICU admission	15 (50.7%)	13 (44.8%)	0.38

CI: confidence interval; CS: caesarean section; ICU: intensive care unit; IUBT: intrauterine balloon tamponade; MD: mean difference; PPH: postpartum hemorrhage; SD: standard deviation; UAE: uterine artery embolization. ^∗^Statistically significant.

**Table 2:** Comparison of the success rate between IUBT and compression sutures in different causes of PPH.

	IUBT	Compression sutures	*p* value
All PPH			
Success	49 (73.1%)	16 (55.1%)	0.15
Additional procedures required	7 (10.5%)	7 (24.1%)	
Hysterectomy	11 (16.4%)	6 (20.7%)	
PPH due to uterine atony			
Success	29 (69.0%)	15 (65.3%)	0.09
Additional procedures required	5 (11.9%)	3 (13.0%)	
Hysterectomy	8 (19.0%)	5 (21.7%)	
PPH due to placenta praevia/accreta			
Success	17 (77.3%)	1 (16.7%)	0.01^∗^
Additional procedures required	2 (9.1%)	4 (66.6%)	
Hysterectomy	3 (13.6%)	1 (16.7%)	

Note: comparing success against additional procedures+hysterectomy. For overall group, *p* = 0.07. For uterine atony, *p* = 0.48. For placenta praevia/accreta, ^∗^*p* = 0.01. IUBT: intrauterine balloon tamponade; PPH: postpartum hemorrhage. ^∗^Statistically significant.

**Table 3:** Risk factors and their association with success rate for uterine sparing procedures.

	a	b	c	*p* value^#^ (a vs. b vs. c)	*b* + *c*	*p* value^†^ (a vs. *b* + *c*); MD (95% CI)
	Success (*n* = 65)	Additional uterine sparing procedures (*n* = 14)	Hysterectomy (*n* = 17)		Additional procedures+hysterectomy (*n* = 31)	
Age	33 (SD 4.5)	33.6 (SD 5)	34 (SD 3.7)	0.65	33.8 (SD 4.3)	0.39; 0.84 (-1.08 to 2.76)
Advanced maternal age (*age* ≥ 35)	40 (61.5%)	8 (57.1%)	8 (47.0%)	0.55	16 (51.6%)	0.24
Parity						
Nulliparous	32 (49.2%)	6 (42.8%)	8 (47.0%)	0.91	14 (45.2%)	0.44
Multiparous	33 (50.8%)	8 (57.2%)	9 (53.0%)		17 (54.8%)	
Gestation at delivery	38.5 (SD 1.5)	37.3 (SD 1.9)	37.7 (SD 1.9)	0.03^∗^	37.5 (SD 1.9)	0.01^∗^; -0.95 (-1.67 to -0.22)
*Preterm* *delivery* < 37 *weeks*	3 (4.6%)	2 (14.2%)	1 (5.9%)	0.40	3 (9.7%)	0.29
Multiple pregnancy	9 (13.8%)	2 (14.2%)	0 (0.0%)	0.56	2 (6.5%)	0.52
Previous CS						
One previous CS	7 (10.7%)	3 (21.4%)	2 (11.8%)	0.19	5 (16.1%)	0.53
Two previous CS	2 (3.0%)	2 (14.2%)	0 (0.0%)		2 (6.5%)	
Mode of delivery						
Normal vaginal	13 (20.0%)	0 (0.0%)	5 (29.4%)	0.33	5 (16.1%)	0.81
Instrumental	2 (3.1%)	1 (7.1%)	0 (0.0%)		1 (3.2%)	
Caesarean	50 (76.9%)	13 (92.9%)	12 (70.6%)		25 (80.6%)	
Aetiology of PPH						
Uterine atony	44 (67.7%)	8 (57.6%)	13 (76.5%)	0.56	21 (67.7%)	0.45
Placenta praevia	8 (12.3%)	6 (42.8%)	4 (23.5%)		10 (32.2%)	
Genital tract trauma	13 (20.0%)	0 (0.0%)	0 (0.0%)		0 (0.0%)	
Blood loss at start of uterine sparing procedure (ml)	1083 (SD 255)	1614 (SD 979)	1782 (SD 475)	<0.001^∗^	1706 (SD 737)	<0.001^∗^; 622 (420 to 825)
*Blood* *loss* < 1.5 *l* at start of uterine sparing procedure	61 (93.8%)	10 (71.4%)	5 (29.4%)	<0.001^∗^	15 (48.3%)	<0.001^∗^
Presence of accreta	1 (1.5%)	0 (0.0%)	5 (29.4%)	<0.001^∗^	5 (16.1%)	0.013^∗^
Coagulopathy	9 (13.8%)	7 (50.0%)	16 (94.1%)	<0.001^∗^	23 (74.2%)	<0.001^∗^
Transfusion of blood products	46 (70.1%)	14 (100.0%)	17 (100.0%)	0.004^∗^	31 (100.0%)	<0.001^∗^
Positive tamponade test (for those with IUBT)	49/49 (100.0%)	6/7 (85.7%)	1/11 (9.1%)	—	7/18 (38.9%)	—
Initial uterine sparing procedure						
IUBT	49 (75.4%)	7 (50.0%)	11 (64.7%)	0.15	18 (58.1%)	0.10
Compression sutures	16 (24.6%)	7 (50.0%)	6 (35.3%)		13 (41.9%)	

CI: confidence interval; CS: caesarean section; IUBT: intrauterine balloon tamponade; MD: mean difference; PPH: postpartum hemorrhage; SD: standard deviation. ^#^*p* value by one-way ANOVA or 3 × 2 contingency table. ^†^*p* value by student *t*-test or chi-square test. ^∗^Statistically significant.

**Table 4:** Logistic regression of prognostic factors against successful management of postpartum hemorrhage by uterine sparing procedures.

Variables in the equation	*B*	Standard error	Wald	*p* value	Odds ratio	95% confidence interval
Blood loss at *procedure* > 1.5 l	1.27	0.44	8.51	0.004	3.57	1.52 to 8.39
Placenta accreta	3.21	1.55	4.35	0.037	24.80	1.21 to 508
Coagulopathy	3.66	0.84	18.90	0.001	38.90	7.48 to 202

## Data Availability

The data is available as Choi Wah Kong; Comparison of the efficacy of intrauterine balloon tamponade and compression sutures; OSF home; DOI 10.17605/OSF.IO/M4AR6.

## References

[1] Hayman R. G., Arulkumaran S., Steer P. J. (2002). Uterine compression sutures: surgical management of postpartum hemorrhage. *Obstetrics and Gynecology*.

[2] Doumouchtsis S. K., Papageorghiou A. T., Arulkumaran S. (2007). Systematic review of conservative management of postpartum hemorrhage: what to do when medical treatment fails. *Obstetrical & Gynecological Survey*.

[3] Bakri Y. N., Amri A., Abdul J. F. (2001). Tamponade-balloon for obstetrical bleeding. *International Journal of Gynecology & Obstetrics*.

[4] Varatharajan L., Chandraharan E., Sutton J., Lowe V., Arulkumaran S. (2011). Outcome of the management of massive postpartum hemorrhage using the algorithm “Hemostasis”. *International Journal of Gynecology & Obstetrics*.

[5] Schnarwyler B., Passweg D., von Castelberg B. (1996). Erfolgreiche behandlung einer medikamentös refraktären uterusatonie durch funduskompressionsnähte. *Geburtshilfe und Frauenheilkunde*.

[6] B-Lynch C., Coker A., Lawal A. H., Abu J., Cowen M. J. (1997). The B-Lynch surgical technique for the control of massive postpartum haemorrhage: an alternative to hysterectomy? Five cases reported. *British Journal of Obstetrics and Gynaecology*.

[7] Matsubara S., Yano H., Ohkuchi A., Kuwata T., Usui R., Suzuki M. (2013). Uterine compression sutures for postpartum hemorrhage: an overview. *Acta Obstetricia et Gynecologica Scandinavica*.

[8] Georgiou C. (2009). Balloon tamponade in the management of postpartum haemorrhage: a review. *BJOG : An International Journal of Obstetrics and Gynaecology*.

[9] Kaya B., Guralp O., Tuten A., Unal O., Celik M. O., Dogan A. (2016). Which uterine sparing technique should be used for uterine atony during cesarean section? The Bakri balloon or the B-Lynch suture?. *Archives of Gynecology and Obstetrics*.

[10] Çetin B. A., Aydogan Mathyk B., Atis Aydin A. (2019). Comparing success rates of the Hayman compression suture and the Bakri balloon tamponade. *The Journal of Maternal-Fetal & Neonatal Medicine*.

[11] Palacios-Jaraquemada J. M. (2011). Efficacy of surgical techniques to control obstetric hemorrhage: analysis of 539 cases. *Acta Obstetricia et Gynecologica Scandinavica*.

[12] Mallappa Saroja C. S., Nankani A., El-Hamamy E. (2010). Uterine compression sutures, an update: review of efficacy, safety and complications of B-Lynch suture and other uterine compression techniques for postpartum haemorrhage. *Archives of Gynecology and Obstetrics*.

[13] Hwu Y. M., Chen C. P., Chen H. S., Su T. H. (2005). Parallel vertical compression sutures: a technique to control bleeding from placenta praevia or accreta during caesarean section. *BJOG : An International Journal of Obstetrics and Gynaecology*.

[14] Li G.-T., Li X.-F., Wu B., Li G. (2016). Longitudinal parallel compression suture to control postopartum hemorrhage due to placenta previa and accrete. *Taiwanese Journal of Obstetrics & Gynecology*.

[15] Ratiu A. C., Crisan D. C. (2018). A prospective evaluation and management of different types of placenta praevia using parallel vertical compression suture to preserve uterus. *Medicine*.

[16] Dabelea V., Schultze P. M., McDuffie R. S. (2007). Intrauterine balloon tamponade in the management of postpartum hemorrhage. *American Journal of Perinatology*.

[17] Brown H., Okeyo S., Mabeya H., Wilkinson J., Schmitt J. (2016). The Bakri tamponade balloon as an adjunct treatment for refractory postpartum hemorrhage. *International Journal of Gynecology & Obstetrics*.

[18] Nelson W. L., O’Brien J. M. (2007). The uterine sandwich for persistent uterine atony: combining the B-Lynch compression suture and an intrauterine Bakri balloon. *American Journal of Obstetrics and Gynecology*.

[19] Diemert A., Ortmeyer G., Hollwitz B. (2012). The combination of intrauterine balloon tamponade and the B-Lynch procedure for the treatment of severe postpartum hemorrhage. *American Journal of Obstetrics and Gynecology*.

[20] Joshi V. M., Shrivastava M. (2004). Partial ischemic necrosis of the uterus following a uterine brace compression suture. *British Journal of Obstetrics and Gynaecology*.

[21] Sentilhes L., Gromez A., Razzouk K., Resch B., Verspyck E., Marpeau L. (2008). B-Lynch suture for massive persistent postpartum hemorrhage following stepwise uterine devascularization. *Acta Obstetricia et Gynecologica Scandinavica*.

[22] Liu S., Mathur M., Tagore S. (2014). Complications and pregnancy outcome following uterine compression suture for postpartum haemorrhage: a single centre experience. *Journal of Obstetrics and Gynaecology*.

[23] Gezginç K., Yazici F., Koyuncu T. (2011). Results of hysterosalpingogram in women with previous B-Lynch suture. *International Journal of Gynaecology and Obstetrics*.

[24] Tadakawa M., Sugawara J., Saito M. (2015). Fertility and pregnancy outcomes following B-Lynch sutures for post-partum hemorrhage. *The Journal of Obstetrics and Gynaecology Research*.

[25] Goojha C. A., Case A., Pierson R. (2010). Development of Asherman syndrome after conservative surgical management of intractable postpartum hemorrhage. *Fertility and Sterility*.

[26] Leparco S., Viot A., Benachi A., Deffieux X. (2013). Migration of Bakri balloon through an unsuspected uterine perforation during the treatment of secondary postpartum hemorrhage. *American Journal of Obstetrics and Gynecology*.

[27] Kong C. W., To W. W. K. (2018). Menstrual and reproductive outcomes after use of balloon tamponade for severe postpartum hemorrhage. *BMC Pregnancy and Childbirth*.

[28] Lodhi W., Golara M., Karangaokar V., Yoong W. (2011). Uterine necrosis following application of combined uterine compression suture with intrauterine balloon tamponade. *Journal of Obstetrics and Gynaecology*.

[29] Beckmann M. M., Chaplin J. (2014). Bakri balloon during cesarean delivery for placenta previa. *International Journal of Gynaecology and Obstetrics*.

[30] Vintejoux E., Ulrich D., Mousty E. (2015). Success factors for Bakri™ balloon usage secondary to uterine atony: a retrospective, multicentre study. *The Australian & New Zealand Journal of Obstetrics & Gynaecology*.

[31] Kong C. W., To W. W. (2018). Prognostic factors for the use of intrauterine balloon tamponade in the management of severe postpartum hemorrhage. *International Journal of Gynaecology and Obstetrics*.

[32] Cho H. Y., Park Y. W., Kim Y. H., Jung I., Kwon J. Y. (2015). Efficacy of intrauterine Bakri balloon tamponade in cesarean section for placenta previa patients. *PLoS One*.

